# Learning from financial rewards and punishments reduces the in-group bias in social approach without changing the in-group bias in impressions

**DOI:** 10.1098/rsos.250061

**Published:** 2025-09-17

**Authors:** Jasper Amadeus Bischofberger, Anne Saulin, Yuqing Zhou, Grit Hein

**Affiliations:** ^1^ Department of Psychiatry, Psychosomatics, and Psychotherapy, University Hospital Würzburg, Würzburg, Germany; ^2^ Centre for Human Brain Health, School of Psychology, University of Birmingham, Birmingham, UK; ^3^ Institute of Psychology, Chinese Academy of Sciences, Beijing, People’s Republic of China

**Keywords:** social, reinforcement learning, approach–avoidance, in-group, out-group

## Abstract

Humans’ approach behaviour and impressions are biased towards individuals from their own group (in-group) compared with different groups (out-group). There is evidence that learning from specific interactions with in-group and out-group members can reduce these in-group biases, but it is yet unclear if learning from non-social reinforcers, such as financial rewards and punishments, can have similar effects. Here, we conducted three independent studies by using different versions of a novel approach–avoidance learning task, intergroup impression ratings and computational learning models. In the approach–avoidance learning task, participants moved a manikin representing themselves towards or away from one of two symbols, representing in-group or out-group individuals or which had no social meaning. Approach was financially rewarded with varying probabilities. Our results confirmed initial in-group biases in approach and impression ratings. Rewarding out-group approach significantly reduced the in-group bias in approach, with stronger learning from rewards compared with punishments. In contrast, the in-group bias in impressions remained unchanged. Two further studies showed that learning-related changes in approach are larger in social compared with non-social contexts and require varying reward probabilities. Together, these findings show that learning from financial rewards or punishments can improve out-group approach but not out-group impressions.

## 1. Introduction

Humans are biased towards individuals of their own group. These in-group biases are found on the behavioural level [1–7] and with regard to subjective impressions of the social group [8–12]. On the behavioural level, there is evidence that stimuli associated with the participant’s social in-group facilitate approach behaviour, whereas out-group stimuli facilitate avoidance behaviour [1–4,7]. While identifying and quantifying in-group approach biases, these previous findings did not yield insights into learning-related, dynamic changes and potential plasticity of these biases.

Regarding impressions, there is ample evidence that individuals form and maintain more positive impressions towards in-group, as compared with out-group individuals [13,14]. In-group biases in impressions are readily elicited not only in ingrained intergroup contexts [15] but also in minimal group paradigms [12]. Recent evidence shows that such impression in-group biases can be reduced by reinforcement learning [9,16–18]. For example, in a recent study, Zhou *et al.* [18] tested how identical, intermixed experiences with different in-group and out-group individuals shape intergroup impressions and perceived closeness to these individuals. Initially, participants showed more positive impression ratings and higher closeness ratings for the in-group compared with the out-group. Learning from identical experiences with both the in-group and the out-group (relief from pain in 75% of trials) significantly reduced the in-group bias in closeness ratings and impression ratings, for the latter with larger effects for participants with stronger in-group identification. Providing insights into the mechanisms, reinforcement learning modelling showed that unexpected in-group and out-group experiences generated a learning signal (prediction error), because they were in conflict with participants’ negative out-group and positive in-group expectations. The weight of these prediction errors accounted for the observed reduction in impression in-group bias, with a particularly strong effect of learning from negative in-group experiences. Other studies used facial stimuli from the same or different ethnicity of the participant to investigate the learning mechanism through which an initial choice bias was updated by trial-by-trial experiences [5,6]. In each trial, participants chose one of two potential interaction partners, who might share a point with them (reward) or not (punishment). Applying computational models of reinforcement learning, they found that an initial in-group bias was updated with separate learning rates for the two different ethnicities (social groups). In a study by Allidina & Cunningham [19], participants also learnt from positive and negative experiences with group members (point allocation versus deduction), but the groups were formed based on artificial entities (‘aliens’) that differed in colour. Using reinforcement learning, the authors showed that participants established an approach bias towards one of the groups, which generalized to new entities that were characterized by random reward probabilities. Moreover, they provide evidence that the decision to avoid increases the likelihood of later avoidance. These learning effects were evident in behaviour (learning-related increase of avoidance), but did not affect participants’ beliefs towards the groups.

Together, these results showed that learning from positive out-group and negative in-group interactions can reduce in-group biases in closeness and impressions, choosing an interaction partner and social approach. However, it remains unknown whether these learning effects generalize to actual in-group and out-group approach behaviour and whether they can be elicited by non-social reinforcers such as financial rewards and punishment, i.e. occur if expected and unexpected experiences with in-group or out-group individuals are replaced by non-social reinforcers such as financial rewards and punishments, instead of the social behaviours. Testing the potential of financial rewards and punishments for unlearning in-group biases is important because these non-social reinforcers are easier to implement and to control than actual in-group and out-group interactions.

There is evidence that financial rewards and punishments efficiently shape behaviour in different domains, including motor skills [20,21] and decision making [22,23]. Many studies show that financial incentives can facilitate behaviour, for example, increase performance levels and the accuracy of decisions [24]. Evidence for an effect of financial rewards and punishments on motivation and attitudes is less consistent [25]. On the one hand, there are studies showing that financial rewards can change intrinsic motivation and even undermine it [26–28]. On the other hand, there is evidence that financial rewards can increase the frequency and efficiency of overt behaviour, without changing participants’ implicit biases [29–31]. Based on these studies, it remains unclear whether and how learning from such non-social outcomes (i.e. financial rewards and punishments that are unrelated to behaviour of others) shapes intergroup approach behaviour and intergroup impressions.

To investigate this question, we used a novel approach–avoidance learning task in combination with reinforcement learning modelling in which participants received financial rewards and punishments for in-group and out-group approach and indicated in-group and out-group impressions. Group membership was manipulated based on nationality (German versus Chinese), one of many possible types to define in-group and out-group. We chose nationality, because it is an ecologically valid manipulation and known to elicit stable intergroup differences [1,2,18,32].

In more detail, in three independent studies, participants moved a manikin (representing themselves) closer to or away from two symbols that represented an in-group or out-group individual (Study 1 and Study 2) or had no social meaning (Study 3). In the social conflict study (Study 1), the financial reward probabilities were higher when approaching the out-group (80%) compared with the in-group (20%), conflicting with the assumed in-group bias in approach. To test the specificity of the potential effects and distinguish them from unspecific changes over time, we designed two control studies (Study 2 and Study 3). In the social control study (Study 2), we used the same social intergroup paradigm but omitted the conflicting financial rewards and used random rewards for in-group and out-group approach instead (50% in both cases). In the non-social control study (Study 3), we applied the same reward probabilities as in Study 1 to a non-social context (i.e. where the symbols had no social meaning) to test if the observed effects reflect general learning effects or are specific for the intergroup context. In each study, participants rated their impressions of the in-group and the out-group before and after learning, allowing us to investigate potential changes in intergroup impressions in the different learning contexts.

Based on the well-established in-group biases in approach [1–4,7] and impressions [13,14,18], we hypothesize that initially participants show a stronger tendency to approach and more positive impressions for the in-group compared with the out-group. Given the assumed positive in-group and negative out-group prior, repeated financial punishments in the in-group context should generate a negative prediction error, and repeated financial rewards in the out-group context should generate positive prediction errors, because these outcomes deviate from the prior expectations. These prediction errors, in turn, should result in a learning-related decrease in in-group approach and a learning-related increase in out-group approach, resulting in an overall decline of the in-group approach bias. Given the previous evidence showing reinforcement learning effects on intergroup impressions [18], it is possible that punishing in-group approach and rewarding out-group approach also reduces the in-group bias in impressions. Based on previous work [18], such learning effects should be stronger in individuals who identify strongly with their in-group. Alternatively, it is possible that financial rewards and punishments induce learning-related changes on the behavioural level, while leaving impressions unchanged, bolstering evidence that financial rewards facilitate behaviour, but do not change biases [19,29–31]. If the hypothesized changes in in-group and out-group approach and intergroup impressions are related to learning, they should be predicted by the individual extent of the learning signal (prediction errors) and not occur if financial rewards and punishments occur randomly, as indicated by larger learning-related changes in Study 1 compared with Study 2. Moreover, if the learning-related changes are specific for unlearning in-group biases in approach and impression, they should not occur if the symbols do not represent in-group and out-group individuals, as reflected by stronger learning-related changes in the social conflict study as compared with the non-social control study.

## 2. Methods

### 2.1. Participants

We conducted three independent studies (*N*_total_ = 144; 76 females, 52.78%; mean age = 28.61, s.d. = 4.59; German nationality; age between 18 and 35 years) via the online platform Clickworker (https://www.clickworker.de). Sample size per study was determined based on the effect size (*d* = 0.444) obtained in a pilot study (https://osf.io/73zxe). Using the software G*Power v. 3.1.9.7 [33], we estimated a sample size of *n* = 42 per study for alpha = 0.05 and a power of 1-beta = 0.8 required for a *t*‐test for the difference between two dependent means. We excluded participants who had more than 5% invalid trials in any block per symbol (i.e. more than one trial), defined as trials in which participants did not move the manikin at all. To account for potential dropouts based on this criterion, we recruited 60 participants in the social conflict study (Study 1), 60 participants in the social control study (Study 2) and 60 participants in the non-social control study (Study 3). After applying the exclusion criteria, we analysed data from 47 participants (26 females, 55.32%; mean age ± s.d. = 29.8 ± 4.3) in Study 1, 50 participants (25 females, 50%; mean age ± s.d. = 28.3 ± 4.6) in Study 2 and 47 participants (25 females, 53.19%; mean age ± s.d. = 27.8 ± 4.7) in Study 3. Participants received monetary compensation of 4€, plus up to 7€, depending on their choices during the experimental task. All participants gave informed consent. The study was approved by the local ethics committee (41/23). The three samples were matched with regard to gender and were comparable with respect to age and questionnaire measures (table 1). For all three studies, we used the same standardized experimental procedure. The studies only differed with regard to one specific experimental condition, which allowed us to compare them with the factor study as a between-subjects variable.

**Table 1.** Sample characteristics of the three studies. The three groups did not differ significantly in any of these measures, suggesting good comparability of the sample characteristics for all three studies. s.d., standard deviation.

	Study 1	Study 2	Study 3	ANOVA	
variables	mean ± s.d.	mean ± s.d.	mean ± s.d.	*F*-value	*p*
age	29.8 ± 4.3	28.3 ± 4.6	27.8 ± 4.7	2.337	0.1
social in-group identification	4.3 ± 1.1	4.3 ± 1.3	4.2 ± 1.1	0.19	0.827
in-group impression (pre)	4.2 ± 1.1	4.2 ± 1.2	4.0 ± 1.2	0.494	0.611
out-group impression (pre)	3.7 ± 1.2	3.5 ± 1.0	3.8 ± 1.1	1.277	0.296
in-group impression (post)	4.1 ± 1.2	4.2 ± 1.2	4.0 ± 1.3	0.283	0.754
out-group impression (post)	3.8 ± 1.1	3.4 ± 1.0	3.8 ± 1.1	2.108	0.125

### 2.2. Procedure

The studies were conducted as pre-registered online studies (https://osf.io/z58xb). We used PsychoPy (v. 2022.2.4) for programming the experimental scripts, including questionnaires, and the online platform Pavlovia for hosting the experiment (https://pavlovia.org). The whole experiment, including questionnaires, took 20−30 min.

#### 2.2.1. Social group induction

Prior to the main experiment, each of the studies started with a social group induction, based on a well-established social priming procedure [18,32,34]. In more detail, participants were asked to list five stereotypical attributes of an individual belonging to their own nationality (in-group; German; ‘Please imagine typical attributes of a person from Germany. Please list the first five characteristics that come to your mind spontaneously.’) or a different nationality (out-group; Chinese; ‘Please imagine typical attributes of a person from China. Please list the first five characteristics that come to your mind spontaneously.’).

Next, participants completed two questionnaires, a validated German version of the in-group identification scale (15 items; 7-point Likert scale ranging from 1 to 7) [35] to quantify their in-group identification and an impression scale (7 items; 7-point Likert scale ranging from 1 to 7) [36] to measure individual impressions of the in-group (German) and the out-group (Chinese). The impression scale was also filled out at the end of the experimental session to test for potential changes in impression ratings from before to after learning.

Participants were introduced to two abstract symbols (geometric diamond and hexagon shape). We specifically chose abstract symbols that should not imply any social meaning, nor any valence at all, allowing us to induce specific associations with these symbols in a controlled way. In the social conflict study and the social control study, participants were instructed that one of the two symbols represented an individual from their social in-group (German nationality), and the other symbol represented an individual from their social out-group (Chinese nationality). The assignment of the symbols to the in-group and out-group was kept constant across the experiment and counterbalanced between participants. After the first and the second of three experimental blocks, participants were reminded of the assignment of the symbols to the social groups. In the non-social control study, we used the same symbols without a social meaning. Using the same symbols in the non-social control study allowed us to compare learning elicited by symbols with identical perceptual features that only differ with regard to whether they carry a social meaning or not.

#### 2.2.2. Approach–avoidance learning task

**Figure 1.**
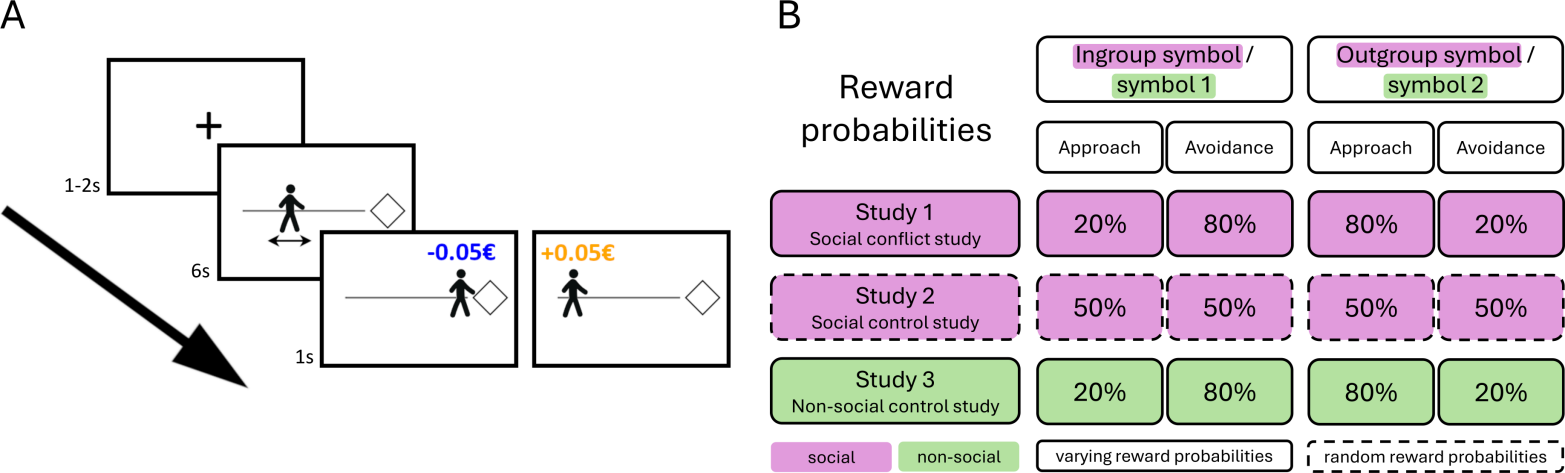
Exemplary trial of the approach–avoidance (AA) learning task and overview of reward probabilities. (A) After a fixation of a jittered length between 1 and 2 s, participants were shown a manikin representing themselves on a horizontal line. On the right side of the line, an abstract symbol was shown (diamond or hexagon shape). During the decision phase, participants had 6 s to move the manikin from the starting position on the scale towards or away from the symbol on the right side and to confirm their final position with a button press. After this confirmation, a caption above the manikin (‘+0.05€’ or ‘−0.05€”) informed participants about the outcome. (B) In the social conflict study (Study 1) and the social control study (Study 2), the symbols represented in-group and out-group individuals (magenta), respectively (counterbalanced across participants). In the non-social control study (Study 3), the symbols had no social meaning (green). Reward probabilities were identical in the social conflict and the non-social control study (solid lines) and random in the social control study (dashed lines).

In the main experiment, participants performed the approach–avoidance learning task shown in figure 1. Each trial started with a fixation cross (1–2 s), followed by a decision phase. During the decision phase, they saw a manikin representing themselves on a horizontal line. The initial position of the manikin was jittered around the centre of that line, ranging from 40 to 60% of its length. Participants were instructed to find their ideal position in relation to an abstract symbol shown on the right end of the line. In the social conflict study (Study 1) and the social control study (Study 2), the symbol represented an ostensible other individual (in-group or out-group), as introduced during the social group induction. In the non-social control study (Study 3), the symbols had no social meaning. The participants could move the manikin continuously to the left (i.e. away from the symbol) and to the right (i.e. towards the symbol), pressing and holding the left and right arrow keys. They were instructed to move the manikin and confirm their chosen position within 6 s. We measured the distance from the starting position to the chosen position as approach (distance > 0) or avoidance (distance < 0) as a continuous variable. We chose a task with a continuous response measure, because we were interested in studying dynamic approach and avoidance behaviour. For this purpose, a continuous measure was shown to provide a robust measure of approach–avoidance behaviour [37]. Additionally, the task is more engaging that way, which is especially important for data quality in online studies [38]. The sign of the distance (distance > 0 = approach; distance < 0 = avoidance) was associated with a financial outcome. Binary reward probabilities were used to reduce task complexity and to allow for successful learning in the majority of our participants. Reward (gain of 0.05€) or punishment (loss of 0.05€) was indicated as a blue or orange caption above the manikin at the final position on the scale (figure 1A). The colours associated with positive or negative outcomes were counterbalanced across participants.

In the social conflict study and the non-social control study, moving the manikin closer to one of the symbols (approach) was associated with a low probability of reward (20% of the trials), while moving the manikin away from that symbol (avoidance) was associated with a high probability of reward (80% of the trials). Moving the manikin closer to the other symbol (approach) was associated with a high probability of reward (80% of the trials), while moving the manikin away from that symbol (avoidance) was associated with a low probability of reward (20% of the trials). Thus, participants either received a reward or a punishment after approaching a symbol. In the social control study, rewards and punishments occurred with random probabilities, i.e. 50% probability of reward independent of symbol and behaviour (figure 1B).

If participants took more than 6 s to confirm their final position, they received the feedback ‘Please confirm pressing the space bar!’. If participants did not move the manikin at all, they received the feedback ‘Please move!’, and the trial was counted as invalid. In both cases, the participants did not get any outcome (i.e. no reward or punishment).

Participants performed 40 trials per block (20 trials per symbol). The order of symbols was pseudo-randomized such that there would be no more than two consecutive trials per symbol. This measure was taken to avoid uncontrolled sequential effects (cf. [39]).

Each participant performed three of these blocks, resulting in a total of 120 decision trials per participant (60 per symbol). Between blocks, participants had a self-paced break and could start the next block with a button press. In the social conflict study (Study 1) and the social control study (Study 2), participants were additionally visually reminded of the assignment of the two symbols to the respective social group, in order to ensure that participants would maintain this association. Moreover, they were informed that they would play an additional game in the end with a member of the social group that they approached more.

In these two studies (Study 1 and Study 2), participants were informed before the main behavioural task that their approach and avoidance behaviour during the task would determine the nationality of the person with whom they would play a second social game. This was done to increase the saliency of the task’s social character. The social game in the end was a one-shot dictator game [40] in which participants could voluntarily give away any amount between 0€ and 1€ (in increments of 10 cents) to an ostensible other participant from the social group that they had approached the most across the whole task. Since each participant performed the dictator game only once, either with an in-group or an out-group individual, the resulting data were unbalanced across participants and, therefore, not analysed. Note that we pre-registered a fourth study, which is not reported here because there were systematic irregularities in the practice trials that may have affected the outcomes.

### 2.3. Analyses

We performed all analyses in RStudio (IDE v. 2023.6.1.524) [41]. All analyses were conducted using R v. 4.3.1 [42].

#### 2.3.1. Sample characteristics across studies

For each demographic measure, we conducted a one-way ANOVA [43] to test if the sample characteristics were comparable across the three studies. For each measure, we report the *F*-value and the *p*‐value.

#### 2.3.2. Regression analyses

We ran linear mixed models (LMMs) using the ‘lme4’ package [44] and estimated the fixed effects using Type 3 Wald chi-square tests of the ‘car’ package [45]. Simple slope analyses were conducted using the emtrends function of the ‘emmeans’ package [46]. Results were visualized with the ‘tidyverse’ package [47] and the ‘ggpubr’ package [48]. All continuous variables in our regressions were *z*-scaled. For each of our models, we included participant as a random intercept. We tried to include random slopes for all within-subjects variables in the models. However, most of the models did not converge. We reported all analyses in which we successfully implemented random slopes in the electronic supplementary material.

First, we performed an LMM analysis to evaluate the social group induction with impression ratings for the in-group and the out-group as a continuous dependent variable. As predictors, we included the within-subjects variable social group (in-group/out-group), the between-subjects variable study (social conflict/social control/non-social control), as well as their interaction. Investigating a potential moderation effect of the social in-group identification, we ran an additional LMM analysis with impression ratings as continuous dependent variable. As predictors, we included the within-subjects variable social group (in-group/out-group), the continuous between-subjects variable social in-group identification, the between-subjects variable study (social conflict, social control and non-social control), as well as their interactions.

Second, we performed an LMM analysis for the data of the first trial with distance as continuous dependent variable. The distance in the first trial was defined as the distance that participants moved the manikin towards (positive values) or away from (negative values) the symbol with respect to their starting position in the respective trial. As fixed effects, we included the within-subjects variable symbol (symbol 1/symbol 2), the between-subjects variable study (social conflict/social control/non-social control), as well as their interaction. As follow-up analyses, we performed an LMM analysis with distance as a continuous dependent variable for each study separately, in which we included symbol (symbol 1/symbol 2) as within-subjects fixed effect. Investigating a potential moderation effect of the social in-group identification, we ran an additional LMM analysis with distance as continuous dependent variable. As predictors, we included the within-subjects variable symbol (symbol 1/symbol 2), the continuous between-subjects variable social in-group identification, the between-subjects variable study (social conflict, social control and non-social control), as well as their interactions.

To test the trial-by-trial influence of rewards and punishments, we performed an LMM analysis with distance as a continuous dependent variable. As predictors, we included the within-subjects variables trial (continuous), symbol, previous outcome for the respective symbol (0 = punishment/1 = reward) and the between-subjects variable study (social conflict/social control/non-social control), as well as their interactions. Additionally, we performed the analogous linear mixed effects model analysis with distance as a continuous dependent variable separately for each study.

#### 2.3.3. Bayesian mixed models

For each study, we fitted two Bayesian mixed models to probe the potential null effects of the social group (in-group/out-group) × time (before learning/after learning) interaction effects on impression ratings. The respective first model included an interaction term between social group and time. The second model included only the main effects of social group and time without their interaction. Both models were fitted using the brms package in R [49], with four Markov chain Monte Carlo chains, 2000 iterations per chain and a warm-up period of 1000 iterations. The priors for the fixed effects were set to a normal distribution with a mean of 0 and a s.d. of 1. To compare the two models, we calculated the Bayes factor (BF) using the bayes_factor() function of the package BayesFactor in R [50] for the first model (social group × time) over the second model (social group + time).

#### 2.3.4. Computational modelling

**Figure 2.**
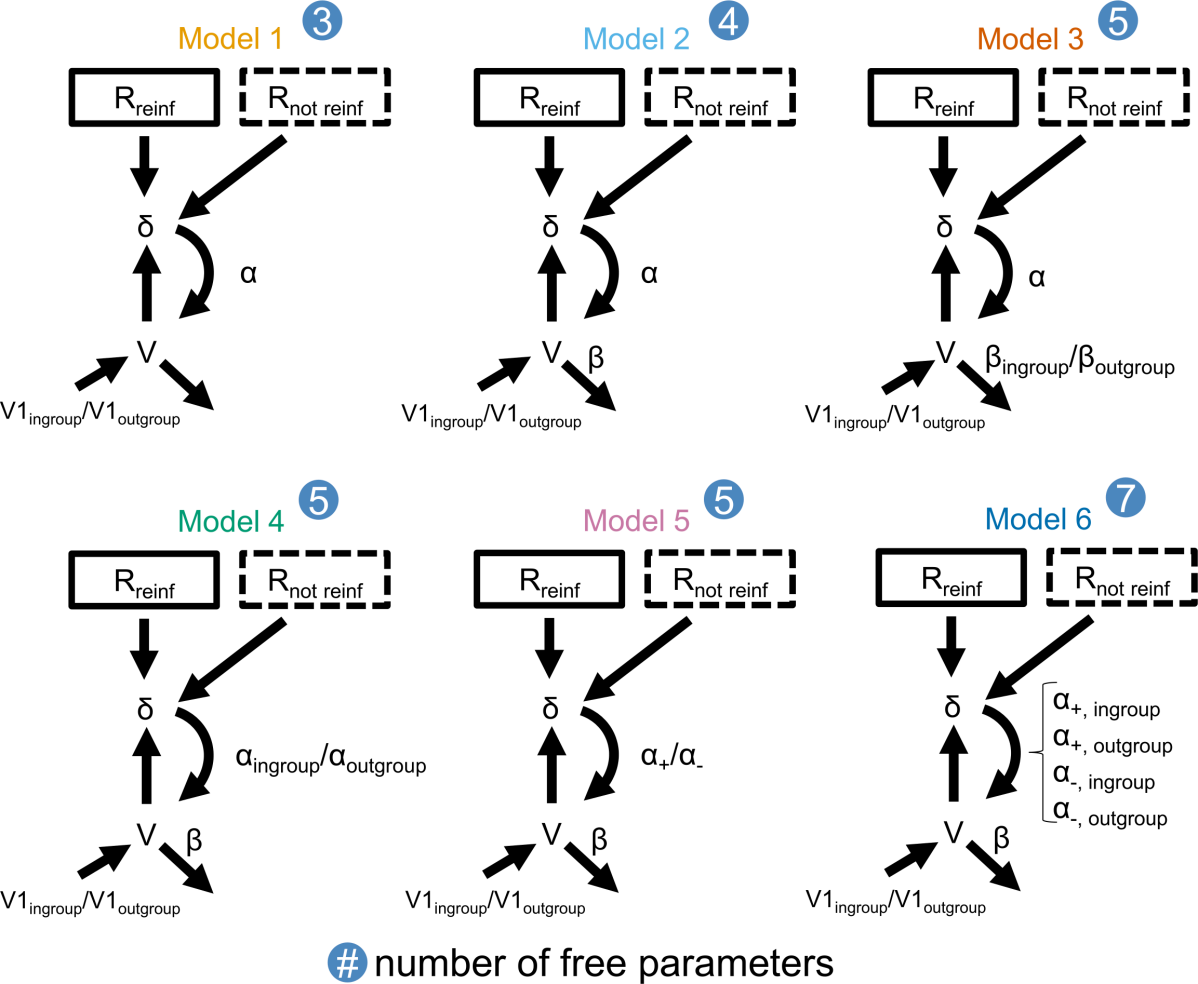
Model space. The figure shows all six models we tested with an increasing number of free parameters. Model 1 (our baseline model) has a single learning rate and two separate starting points for the in-group and out-group symbols (symbol 1 and symbol 2 in the non-social control study). In model 2, we included a response parameter β, accounting for the accuracy with which participants translate a (hidden) expected value into their chosen (observable) distance. In model 3, we included two separate response parameters β for the in-group and out-group symbols (symbol 1 and symbol 2). For model 4, we extended model 2 by two separate learning rates for the in-group and out-group symbols (symbol 1 and symbol 2). For model 5, we extended model 2 by two separate learning rates for positive and negative prediction errors. For model 6, we extended model 2 by four separate learning rates for positive and negative prediction errors for the in-group and out-group symbols (symbol 1 and symbol 2).

To investigate how financial incentives moderate approach and avoidance learning, we used computational models of reinforcement learning to analyse the behavioural data. In detail, we tested which out of six models best captured participants’ behaviour over time (see visualization in figure 2).

In our learning model 1 (equation 2.1), the expected value *V* (participant’s choice = distance) in the present trial *t* is updated according to the Rescorla–Wagner learning rule [51] with the learning rate *α.*


(2.1) V(t)=V(t−1)+α×δ(t),

with the prediction error


(2.2) δ(t)=r(t)–V(t−1).

Thus, the prediction error *δ*(*t*) is computed for each trial *t* as the difference between the observed outcome *r*(*t*) (reward or punishment) and the distance in the previous trial *V*(*t* − *1*). To match the orientation of the reward dimension, we inverted the distance in trials with the in-group symbol (symbol 1 in the non-social control study).

In addition to the learning rate *α*, all our computational models included two independent free parameters *V*1_in-group_ and *V*1_out-group_, reflecting potential initial biases of the in-group and out-group symbols (symbol 1 and symbol 2 in the non-social control study). Thus, this parameter accounts for a hypothesized initial in-group approach bias.

In our learning model 2 (equation 2.3), we included an additional free response parameter *β*, accounting for the weight with which participants translate a (hidden) expected value into their chosen (observable) distance [18].


(2.3) V(t)=[V(t−1)+α×δ(t)]×β.

Learning model 3 (equations 2.4a and 2.4b) is the same as learning model 2, but instead of a single response parameter *β*, we included two independent response parameters for the two social groups *β*_in-group_ and *β*_out-group_ (symbol 1 and symbol 2 in the non-social control study).


(2.4a) $$V(t)=[V(t-1)+\alpha \times \delta (t)]\times \beta_{in-group}$$

(2.4b) $$V(t)=[V(t-1)+\alpha \times \delta (t)]\times \beta_{out-group}.$$

If this model won, this would indicate that participants translate the hidden expected value with specific weight into their chosen distance, depending on the social group or symbol.

Learning model 4 (equations 2.5a and 2.5b) is the same as learning model 2, but instead of a single learning rate *α*, here we included two independent free parameters for the learning rate for updating the expected value for the two social groups *α*_in-group_ and *α*_out-group_ (symbol 1 and symbol 2 in the non-social control study).


(2.5a) $$V(t)=[V(t-1)+\alpha_{in-group}\times \delta (t)]\times \beta$$

(2.5b) $$V(t)=[V(t-1)+\alpha_{out-group}\times \delta (t)]\times \beta .$$

If this model won, this would indicate that the degree to which participants update their expected value according to the prediction error would differ, depending on the social group or symbol.

Learning model 5 (equations 2.6a and 2.6b) is the same as learning model 2, but instead of a single learning rate *α*, we included two independent free parameters for the learning rate for positive prediction errors *δ*(*t*) ≥ 0 (*α*_+_) and negative prediction errors *δ*(*t*) < 0 (*α*_−_).


(2.6a) $$V(t)=[V(t-1)+\alpha_{+}\times \delta (t)]\times \beta \;\;if\delta (t)\geq 0$$

(2.6b) $$V(t)=[V(t-1)+\alpha_{-}\times \delta (t)]\times \beta \;\;if\delta (t)<0.$$

If this model won, this would indicate that the degree to which participants update their expected value would differ for positive and negative prediction errors.

Learning model 6 (equations 2.7a–2.7d) is the same as learning model 2, but instead of a single learning rate *α*, here we included four independent free parameters for the learning rate for updating the expected value in in-group trials for positive prediction errors *δ*(*t*) ≥ 0 (*α*_+, in-group_) or negative prediction errors *δ*(*t*) < 0 (*α*_−, in-group_) or in out-group trials for positive prediction errors δ(t) ≥ 0 (α_+, out-group_) or negative prediction errors δ(t) < 0 (α_−, out-group_).


(2.7a) $$V(t)=[V(t-1)+\alpha_{+},{\;}_{in-group}\times \;\delta (t)]\times \beta \;\;if\;\delta (t)\geq 0$$

(2.7b) $$V(t)=[V(t-1)+\alpha_{+},{\;}_{out-group}\times \;\delta (t)]\times \beta \;\;if\;\delta (t)\geq 0$$

(2.7c) $$V(t)=[V(t-1)+\alpha_{-},{\;}_{in-group}\times \;\delta (t)]\times \beta \;\;if\;\delta (t)<0$$

(2.7d) $$V(t)=[V(t-1)+\alpha_{-},{\;}_{out-group}\times \;\delta (t)]\times \beta \;\;if\;\delta (t)<0.$$

If this model won, this would indicate that the degree to which participants update their expected value would differ for positive and negative prediction errors and the social group or symbol.

Fitting participants’ choice data to these computational models, we aim to disentangle learning mechanisms of approaching and avoiding the in-group versus the out-group.

##### 2.3.4.1. Parameter estimation and model comparison

To fit the parameters of the different computational models, we determined the set of parameters that minimized the sum of the squared errors (SSE) between participants’ choices and model predictions given the specific model. We estimated the parameters separately for each participant using the bound constraint Broyden–Fletcher–Goldfarb–Shanno optimization algorithm [52]. We initialized the optimization function with randomly selected start values between 0 and 1 to prevent local minima. Model implementations and parameter fitting were done in R v. 4.3.1. We compared the models using the Bayesian information criterion (BIC) using the formula [53,54],


(2.8) BIC=n×ln(SSE/n)+k×ln(n).

Here, *n* is the number of observations, and *k* is the number of free parameters. BIC measures are summed across all participants. A smaller BIC indicates a better model fit. For testing significant differences between the model parameters, we ran Welch two-sample *t*-tests.

## 3. Results

### 3.1. Manipulation checks

To assess the success of our social group induction, we analysed impression ratings prior to the learning task and the distance towards or away from the two symbols in the first trial.

#### 3.1.1. Impression ratings

First, we analysed participants’ in-group and out-group impression ratings before the approach–avoidance learning task using an LMM with impression rating for the in-group and the out-group as a continuous dependent variable and a random intercept per participant. As predictors, we included the within-subjects variable social group (in-group/out-group), the between-subjects variable study (social conflict, social control and non-social control), as well as their interaction. We found a significant main effect of social group with more negative impression ratings for the out-group as compared with the in-group (*χ*^2^(1) = 5.82, *p* = 0.016, *β*_out-group_ = −0.42, s.e. = 0.17, *t* = −2.41; see table 1 for average values). The main effect of study (*χ*^2^(2) = 1.07, *p* = 0.586, *β*_social control_ = 0.04, s.e. = 0.20, *t* = 0.208, *β*_non-social control_ = −0.16, s.e. = 0.20, *t* = −0.77) and the social group × study interaction (*χ*^2^(2) = 4.18, *p* = 0.124, *β*_out-group, social control_ = −0.22, s.e. = 0.24, *t* = −0.90, *β*_out-group, non-social control_ = 0.28, s.e. = 0.25, *t* = 1.13) were not significant. These findings indicate an in-group bias in impression ratings prior to the learning task that is comparable across studies.

In an additional analysis, we added social in-group identification to test the relationship between initial impression ratings and social in-group identification scores. The results revealed a significant main effect of social group (*χ*^2^(1) = 7.21, *p* = 0.007, *β*_out-group_ = −0.40, s.e. = 0.15, *t* = −2.69) and a significant interaction effect of social group × social in-group identification (*χ*^2^(1) = 27.56, *p* < 0.001, *β*_out-group_ = −0.84, s.e. = 0.16, *t* = −5.25) that was not significantly different between studies (*χ*^2^(2) = 3.81, *p* = 0.149, *β*_social control_ = 0.20, s.e. = 0.21, *t* = 0.97, *β*_non-social control_ = 0.43, s.e. = 0.22, *t* = 1.95), reflecting more negative impression ratings for the out-group compared with the in-group with increasing social in-group identification scores across studies.

#### 3.1.2. Distance in the first trial

**Figure 3.**
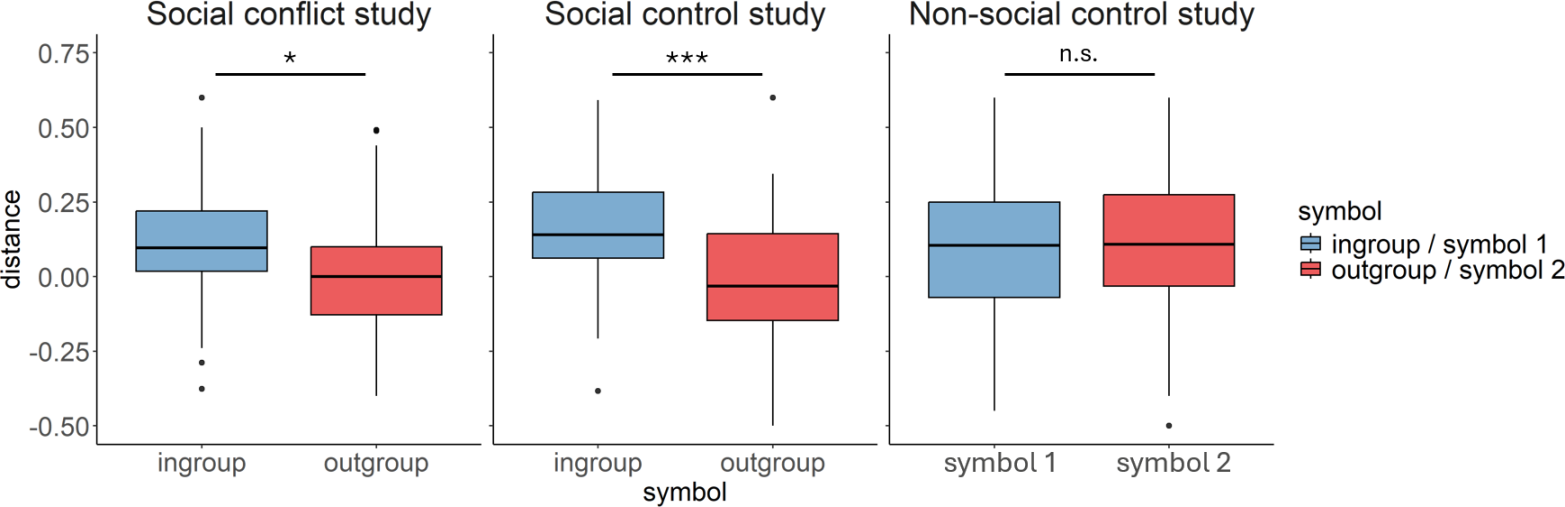
Distance participants moved the manikin towards or away from the symbol in the first trial for the social conflict study (Study 1; left panel), the social control study (Study 2; middle panel) and the non-social control study (Study 3; right panel). Positive distance values indicate moving towards (approaching) the symbol on the right side of the scale, and negative distance values indicate moving away from (avoiding) this symbol. The boxplots show the interquartile range (IQR) from the first quartile (Q1) to the third quartile (Q3), the median indicated by a solid line within the box, upper whiskers extending to 1.5 × IQR above Q3 and lower whiskers extending to 1.5 × IQR below Q1 and outliers as single dots. If the symbols represented an in-group and out-group individual (Studies 1 and 2), participants showed an approach bias towards the in-group, which was not evident if the symbols had no social meaning (Study 3).

Second, based on the data from the first trial, we performed an LMM with distance as continuous dependent variable and a random intercept per participant. As predictors, we included the within-subjects variable symbol (symbol 1/symbol 2), the between-subjects variable study (social conflict, social control and non-social control), as well as their interaction. The results revealed a significant symbol × study interaction (*χ*^2^(2) = 8.01, *p* = 0.018, *β*_out-group, social control_ = −0.25, s.e. = 0.28, *t* = −0.88, *β*_out-group, non-social control_ = 0.53, s.e. = 0.29, *t* = 1.87). To unpack this interaction effect, we performed LMM analyses with distance as a continuous dependent variable and symbol as fixed effect, separately for each study. In the social conflict study (Study 1) and the social control study (Study 2), the initial distance towards the out-group symbol was more negative than towards the in-group symbol (Study 1: *χ*^2^(1) = 6.29, *p* = 0.012, *β*_out-group_ = −0.50, s.e. = 0.20, *t* = −2.51; Study 2: *χ*^2^(1) = 16.54, *p* < 0.001, *β*_out-group_ = −0.76, s.e. = 0.19, *t* = −4.07). In the non-social control study, there was no significant difference in initial distance towards the two symbols (*χ*^2^(1) = 0.11, *p* = 0.741, *β*_symbol 2_ = 0.07, s.e. = 0.21, *t* = 0.33; figure 3). We also tested if the in-group bias in initial distance in the social conflict study (Study 1) and the social control study (Study 2) was related to individual differences in social in-group identification, with no significant effects (Study 1: *χ*^2^(1) = 0.16, *p* = 0.692, *β*_out-group_ = −0.08, s.e. = 0.20, *t* = −0.40; Study 2: *χ*^2^(1) = 0.86, *p* = 0.353, *β*_out-group_ = −0.17, s.e. = 0.19, *t* = −0.93). These results demonstrate the existence of an in-group bias in approach behaviour if the symbols represented an in-group or out-group individual. If the same symbols had no social meaning, the in-group bias would not be evident.

### 3.2. Approach–avoidance learning task

**Figure 4.**
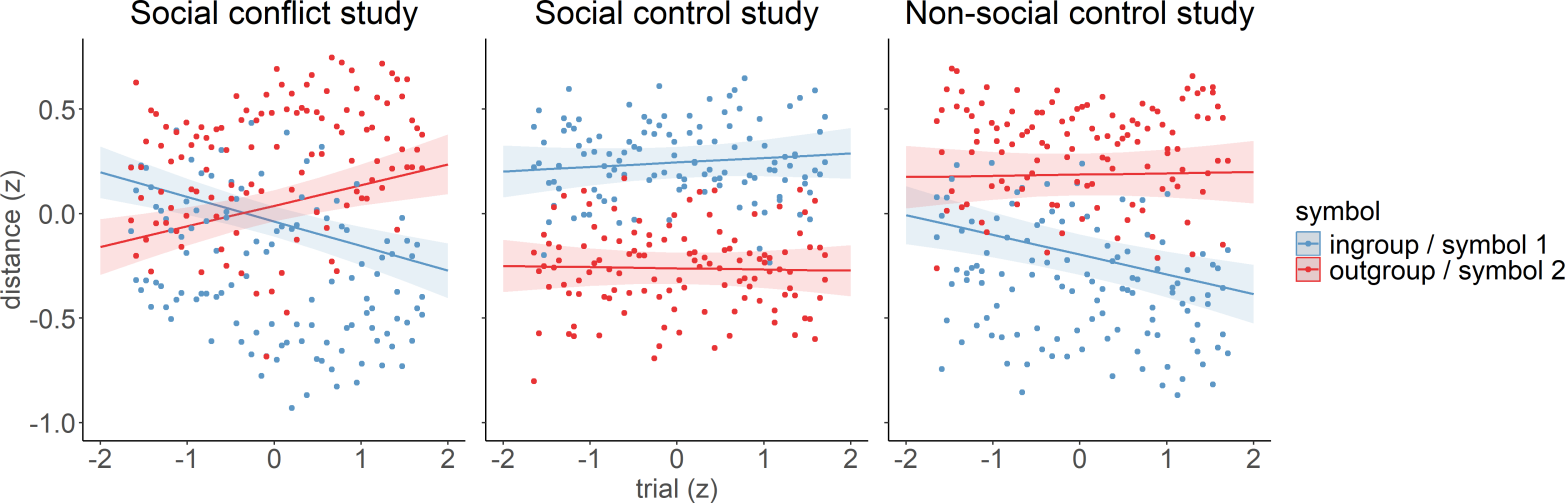
Trial-by-trial changes in distance for the different symbols for the social conflict study (Study 1; left panel), the social control study (Study 2; middle panel) and the non-social control study (Study 3; right panel). Positive distance values indicate moving towards (approaching) the symbol on the right side of the scale, and negative distance values indicate moving away from (avoiding) this symbol. In the social conflict study and the social control study, one symbol was associated with an in-group individual (blue), and the other symbol was associated with the out-group individual (red). In the non-social control study, the two symbols had no social meaning. All variables were *z*-scaled. The plot shows the regression lines (and confidence intervals) of the LMM analysis, overlaid by the aggregated raw data points.

Next, we tested whether and how this in-group bias in social approach is altered by the outcomes during the approach–avoidance learning task, i.e. financial rewards and punishments. We performed an LMM analysis with distance as a continuous dependent variable and trial, previous outcome, symbol, study and their interactions as fixed effects. The results revealed a significant trial × symbol × study interaction effect (*χ*^2^(2) = 20.16, *p* < 0.001, *β*_out-group, social control_ = −0.23, s.e. = 0.05, *t* = −4.39, *β*_out-group, non-social control_ = −0.09, s.e. = 0.06, *t* = −1.55), indicating a learning-related change over time with differential effects between the two symbols and the three studies (figure 4).

To clarify the three-way interaction, first, we compared the slopes of the trial-by-trial changes across studies. The results showed that the slope for approaching the out-group symbol (symbol 2; red) was significantly steeper in the social conflict study compared with the two other studies (*β*_social conflict − social control_ = 0.08, s.e. = 0.025, *z* = 3.232, *p* = 0.016, *β*_social conflict − non-social control_ = 0.08, s.e. = 0.026, *z* = 2.988, *p* = 0.034). There was no significant difference between the social control and the non-social control study (*β*_social control – non-social control_ = −0.003, s.e. = 0.025, *z* = −0.133, *p* = 1.000; figure 4).

The slope for changing the distance towards the in-group symbol (symbol 1; blue) differed significantly between the social conflict study and the social control study (*β*_social conflict − social control_ = −0.11, s.e. = 0.025, *z* = −4.661, *p* < 0.001) and between the social control study and the non-social control study (*β*_social control − non-social control_ = 0.11, s.e. = 0.024, *z* = 4.453, *p* < 0.001). No difference was observed between the social conflict study and the non-social control study (*β*_social conflict − non-social control_ = −0.005, s.e. = 0.025, *z* = −0.211, *p* = 0.999; figure 4).

Note that we ran an additional LMM analysis, including simple random slopes for all within-subjects variables (see electronic supplementary material, results, approach–avoidance learning task, for details).

In the second step, we compared the behaviour towards the in-group and out-group symbols (symbol 1 and symbol 2) within each study.

In the social conflict study, simple slope analyses revealed a significant positive slope for approaching the out-group symbol and a significantly negative slope for avoiding the in-group symbol (*β*_out-group_ = 0.09, s.e. = 0.02, 95% CI (0.05,0.13), *β*_in-group_ = −0.11, s.e. = 0.018, 95% CI (−0.15, −0.08)). Consequently, the slope for approaching the out-group symbol was significantly more positive than the slope for avoiding the in-group symbol (*β*_in-group − out-group_ = −0.202, s.e. = 0.026, *z* = −7.927, *p* < 0.001; figure 4).

In the social control study, simple slope analyses did not show a significant slope for approaching/avoiding the in-group symbol nor a significant slope for approaching/avoiding the out-group symbol (*β*_in-group_ = 0.008, s.e. = 0.018, 95% CI (−0.027,0.043), *β*_out-group_ = 0.004, s.e. = 0.018, 95% CI (−0.031, 0.039)). The slope for approaching/avoiding the in-group symbol did not significantly differ from the slope for approaching/avoiding the out-group symbol (*β*_in-group − out-group_ = 0.004, s.e. = 0.025, *z* = 0.164, *p* = 0.870; figure 4).

In the non-social control study, simple slope analyses did not show a significant slope for approaching/avoiding symbol 2, but a significantly negative slope for avoiding symbol 1 (*β*_symbol 2_ = 0.006, s.e. = 0.018, 95% CI (−0.029,0.040), *β*_symbol 1_ = −0.088, s.e. = 0.017, 95% CI (−0.12, −0.054)). The slope for approaching symbol 2 was significantly larger than the slope for avoiding symbol 1 (*β*_symbol 1 – symbol 2_ = −0.093, s.e. = 0.024, *z* = −3.786, *p* < 0.001; figure 4).

#### 3.2.1. Effects of in-group identification

The results reported above indicate that participants decrease their avoidance of the out-group and also their approach of the in-group if they are incentivized to do so. We hypothesized that these effects may be moderated by in-group identification. To test this, we included the social in-group identification score as an additional continuous between-subjects variable into the LMM above. We found a significant four-way interaction (social in-group identification × trial × symbol × study (*χ*^2^(2) = 11.08, *p* = 0.004, *β*_out-group, social control_ = −0.15, s.e. = 0.05, *t* = −2.72, *β*_out-group, non-social control_ = −0.19, s.e. = 0.06, *t* = −3.17).

To clarify this interaction, we ran the same LMM separately for each study. In the social conflict study, we found a significant three-way interaction effect of trial × symbol × social in-group identification (*χ*^2^(1) = 18.43, *p* < 0.001, *β*_out-group_ = 0.17, s.e. = 0.04, *t* = 4.29), reflecting an increase in out-group approach and in-group avoidance with increasing in-group identification scores (*χ*^2^(1) = 46.75, *p* < 0.001, *β*_out-group_ = 0.27, s.e. = 0.04, *t* = 6.84). There were no significant trial × symbol × social in-group identification interaction effects in the social control study (*χ*^2^(1) = 1.35, *p* = 0.246, *β*_out-group_ = 0.03, s.e. = 0.03, *t* = 1.16) and the non-social control study (*χ*^2^(1) = 0.11, *p* = 0.743, *β*_symbol 2_ = −0.01, s.e. = 0.04, *t* = −0.33).

### 3.3. Changes in impression ratings before and after learning

**Figure 5.**
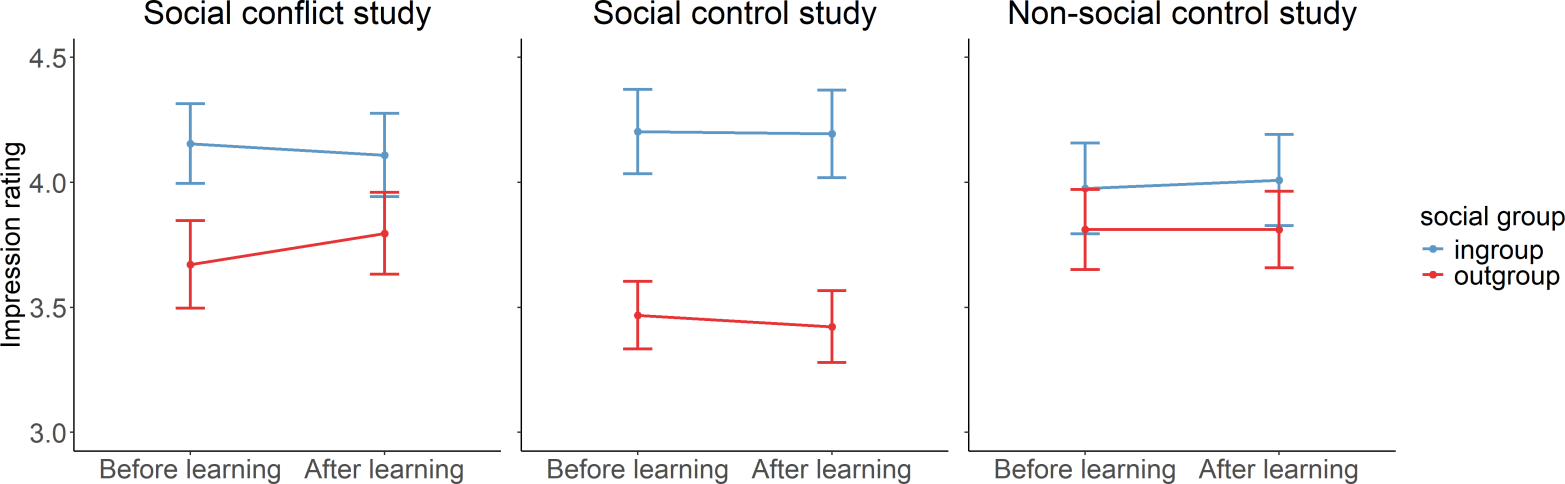
Impression ratings before and after the approach–avoidance learning task for the social conflict study (Study 1; left panel), the social control study (Study 2; middle panel) and the non-social control study (Study 3; right panel). The line graphs show the mean value (±s.e.). All variables were *z*-scaled. The results show that in-group and out-group impression ratings did not significantly change from before to after learning.

The results revealed that financial incentives and punishments incite learning-related changes in intergroup social approach, dissolving the initial in-group approach bias. We tested whether such changes also occur in intergroup impressions. To do so, we performed an LMM analysis with impression rating as a continuous dependent variable and the fixed effects social group (in-group/out-group), time (before learning/after learning), study (social conflict, social control, non-social control) and their interactions. The model revealed a significant main effect of social group (*χ*^2^(1) = 8.12, *p* = 0.004, *β*_out-group_ = −0.42, s.e. = 0.15, *t* = −2.85; figure 5), suggesting an in-group impression bias before and after the approach–avoidance learning task. However, the interaction effects were not significant (social group × time (*χ*^2^(1) = 0.50, *p* = 0.478, *β*_out-group, post_ = 0.15, s.e. = 0.21, *t* = 0.71; social group × time × study (*χ*^2^(2) = 0.50, *p* = 0.781, *β*_out-group, after learning, social control_ = −0.18, s.e. = 0.29, *t* = −0.62, *β*_out-group, after learning, non-social control_ = −0.18, s.e. = 0.29, *t* = −0.60), indicating a persistence of the in-group bias in impression ratings, irrespective of the approach–avoidance learning task in all studies (figure 5).

To follow up on this lack of significance, for each study, we fitted two Bayesian mixed models. The first model included an interaction term between social group and time, i.e. social group × time. The second model included only the main effects of social group and time without their interaction, i.e. social group + time. To compare the two models, we calculated the BF for the first model (social group × time) over the second model (social group + time). For each study, we found that the respective model including the interaction was less likely than the respective model without the interaction (social conflict study: BF_10_ = 0.2609; social control study: BF_10_ = 0.1997; non-social control study: BF_10_ = 0.1757). This evidence suggests that the inclusion of the interaction term between social group and time does not meaningfully improve model fits compared with the simpler models that include only main effects in all three studies. This finding corroborates the evidence that the impression ratings do not change from before to after learning.

### 3.4. Computational modelling

We used reinforcement learning modelling based on the Rescorla–Wagner learning rule [51] to investigate the underlying mechanisms of the significant trial-by-trial changes that we observed in the social conflict and the non-social control studies (figure 4, left and right panels). Note that we refrained from modelling the social control study because there were no significant trial-by-trial changes, indicating a lack of learning (figure 4, middle panel).

We fitted the different versions of the reinforcement learning models that we specified in §2 to participants’ individual data from the social conflict and the non-social control studies.

**Figure 6.**
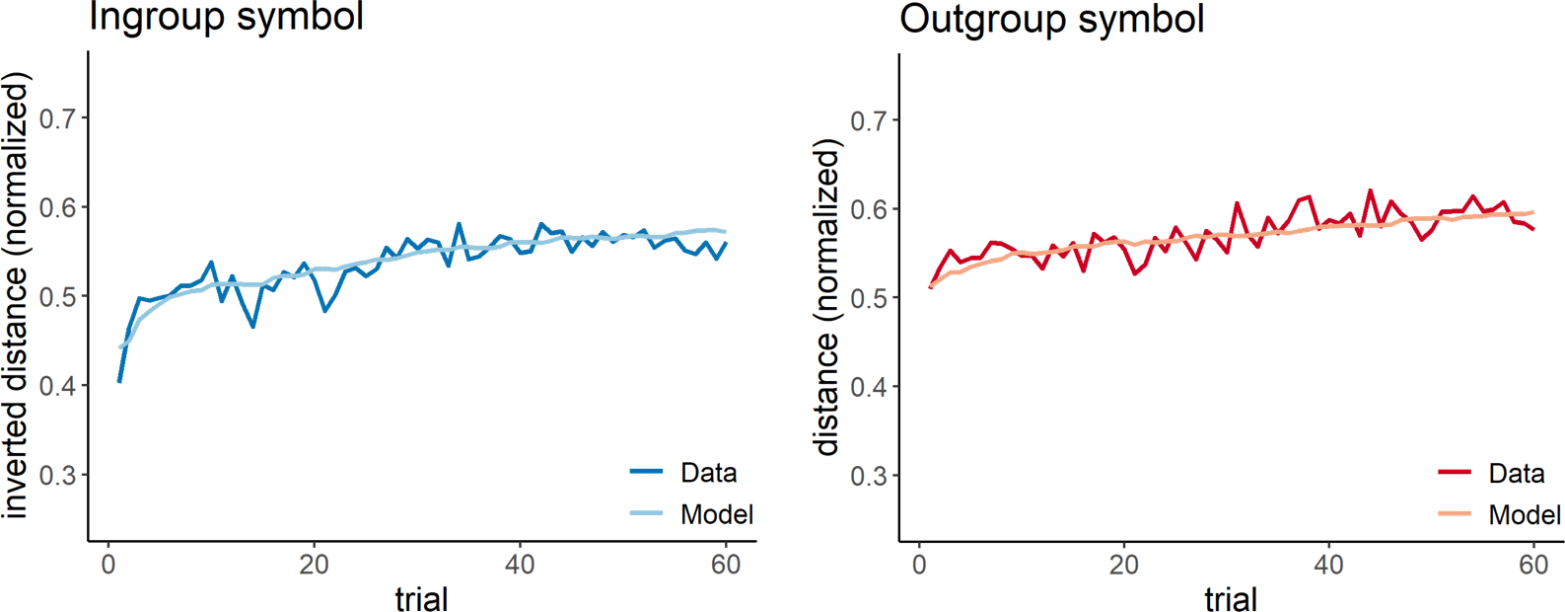
Model predictions of the winning model (Model 5) of the social conflict study. The plots show the distance towards (i.e. approach) or away from (i.e. avoidance) the in-group symbol (blue) and the out-group symbol (red) across the trials in the experiment. Each plot shows the averaged raw data (dark colour) and the corresponding model predictions (light colour). The distance is normalized between 0 and 1. For the in-group symbol, the distance is inverted to match the orientation of the reward dimension.

In the social conflict study, model comparison revealed the best model fit for Model 5 (table 2), including two separate learning rates for trials with positive and negative prediction error, two initial distances for in-group and out-group and a single response parameter (*R*^2^ = 0.21 ± 0.18; mean ± s.d.; figure 6 for model predictions). The learning rate for positive prediction errors (*α*_+_) was significantly higher than the learning rate for negative prediction errors (*α*_−_) (*t* = 6.40, *df* = 46.18, *p* < 0.001, *α*_+_ = 0.38 ± 0.39, *α*_−_ = 0.01 ± 0.02; mean ± s.d.). The initial distance for the in-group (*V*1_in-group_) was significantly smaller than for the out-group (*V*1_out-group_) (*t* = −2.22, *df* = 90.68, *p* = 0.029, *V*1_in-group_ = 0.70 ± 0.24, *V*1_out-group_ = 0.80 ± 0.21; mean ± s.d.). The estimate for the response parameter was *β* = 0.67 ± 0.18 (mean ± s.d.).

**Table 2.** Comparisons of model fits for distance for the social conflict study. BIC measures are summed across all participants. Lower BIC values indicate better model fit. Mean squared error (*R*^2^) over distance indicates goodness of fit. Importantly, in contrast to *R*^2^, the BIC measures take the number of free parameters of the model (*K*) into account (cf. equation 2.8). The winning model is highlighted with bold font.

	*K*	*R* ^2^	BIC
model 1	3	0.11	−27 552
model 2	4	0.12	−27 415
model 3	5	0.13	−27 230
model 4	5	0.13	−27 276
**model 5**	**5**	**0.21**	**−27 904**
model 6	7	0.24	−27 703

**Figure 7.**
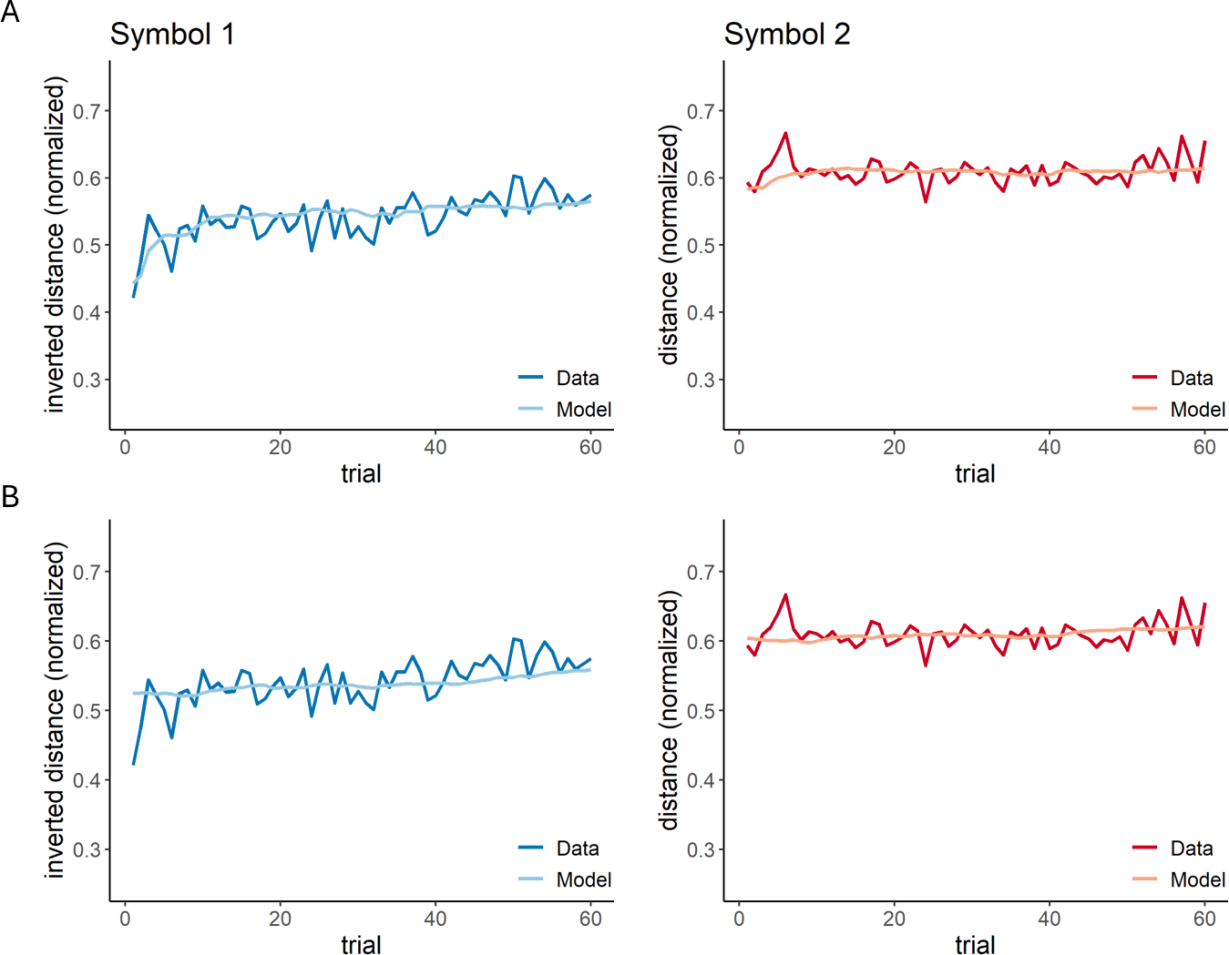
Model predictions of (A) Model 5 and (B) Model 1 of the non-social control study. The plots show the distance towards (i.e. approach) or away from (i.e. avoidance) symbol 1 (blue) and symbol 2 (red) across the trials in the experiment. Each plot shows the averaged raw data (dark colour) and the corresponding model predictions (light colour). The distance is normalized between 0 and 1. For symbol 1, the distance is inverted to match the orientation of the reward dimension.

Model 5 also accounted reasonably well for learning-related changes in the non-social control study (*R*^2^ = 0.17 ± 0.2; mean ± s.d.; figure 7A for model predictions) with similar model parameters as in the social conflict study (*V*1_in-group_ = 0.67 ± 0.26, non-social control versus social conflict: *t* = 0.52, *df* = 91.71, *p* = 0.602; *V*1_out-group_ = 0.88 ± 0.19, non-social control versus social conflict: *t* = −1.80, *df* = 90.98, *p* = 0.076; *α*_+_ = 0.36 ± 0.39, non-social control versus social conflict: *t* = 0.16, *df* = 91.98, *p* = 0.870; α_−_ = 0.01 ± 0.02, non-social control versus social conflict: *t* = −0.62, *df* = 86.82, *p* = 0.539; *β* = 0.68 ± 0.17, non-social control versus social conflict: *t* = −0.22, *df* = 91.55, *p* = 0.828; mean ± s.d.). However, with regard to the BIC values (i.e. the value that takes into account the number of free model parameters required to fit the data), Model 5 was outperformed by the most basic model that just contained a single learning rate and two initial distances for symbol 1 and symbol 2 (Model 1: *R*^2^ = 0.12 ± 0.21; *V*1_symbol 1_ = 0.53 ± 0.14, *V*1_symbol 2_ = 0.53 ± 0.14, *α* = 0.01 ± 0.02; mean ± s.d.; figure 7B for model predictions; table 3 for model comparison). This indicates that a simple model is sufficient to account for learning-related differences in approaching or avoiding abstract symbols without social meaning. This finding suggests that the underlying learning mechanisms are less complex as compared with the social conflicting context.

**Table 3.** Comparisons of model fits for distance for non-social control study. BIC measures are summed across all participants. Lower BIC values indicate better model fit. Mean squared error (*R*^2^) over distance indicates goodness of fit. Importantly, in contrast to *R*^2^, the BIC measures take the number of free parameters of the model (*K*) into account (cf. equation 2.8). The winning model is highlighted with bold font.

	*K*	*R* ^2^	BIC
**model 1**	**3**	**0.12**	**−23 078**
model 2	4	0.13	−22 888
model 3	5	0.13	−22 683
model 4	5	0.13	−22 712
model 5	5	0.17	−22 998
model 6	7	0.19	−22 669

## 4. Discussion

Humans show in-group biases in overt behaviour and with regard to covert impressions and attitudes. Here we tested whether repeated financial rewards and punishment can change in-group biases on these different levels, i.e. with regard to approach behaviour and intergroup impressions. Our results showed that financial rewards in an out-group context and financial punishments in an in-group context significantly reduced the in-group bias in approach behaviour that participants showed before learning (social conflict study). When participants received the same financial rewards and punishments randomly (social control study), they did not change their intergroup behaviour, indicating that the in-group approach bias remains stable over time without a reinforcement scheme eliciting learning. In more detail, when receiving financial reward for out-group approach in the social conflict study, participants dynamically increased their approach towards a symbol representing an out-group individual. When receiving financial punishment for approaching the in-group, they started to avoid the in-group symbol. The individual extent of this learning-related increase in out-group approach and decrease in in-group approach was linked to individual differences in in-group identification. The stronger a person identified with the in-group that was represented by the symbol, the stronger the increase in out-group approach following financial reward and decrease in in-group approach following financial punishment. Although participants learnt to approach the out-group and to avoid the in-group, they did not significantly change the in-group bias in impressions. This indicates that financial incentives can dynamically shape individuals’ behaviour towards out-group members, but do not improve their relative out-group impressions.

Our finding that financial rewards and punishments influence overt behaviour, but not intergroup impressions, is in line with previous findings showing that reward increases the frequency and efficiency of a particular behaviour, without changing the pre-existing bias. For example, studies using drift-diffusion modelling showed that financial rewards increased participants’ frequency and efficiency (indicated by the drift rate) of prosocial behaviour but did not change their initial prosocial decision bias (indicated by the *z*-parameter) [29]. In the social domain, they converge with evidence of other previous studies that investigated social approach using abstract entities (e.g. blue- and green-skinned aliens) that were associated with a social meaning (e.g. a cooperative or non-cooperative group; [19]). As in our study, participants learnt to establish an approach bias towards one of the groups, but did not change participants’ beliefs towards this group. In contrast to our study, these learning effects were based on actual experiences with the abstract entities, i.e. the aliens allocating or deducting a point when approached by the participant. Extending this previous research, we show that learning-related changes in social approach can be elicited by non-social reinforcers whose value contradicts the social bias.

In more detail, our learning models show that individuals change their intergroup approach behaviour based on a prediction-error signal that was generated while receiving financial rewards and punishments. Specifying the underlying mechanism, participants’ changes in behaviour in the social conflict study were best captured by a model that differentiated between positive and negative learning rates, indicating that participants learnt differently from financial rewards and punishments. In more detail, participants showed a higher positive learning rate, indicating that they learnt faster from rewards compared with punishments. The finding that prediction-error-related learning increased out-group and decreased in-group approach is in line with previous findings showing a change in intergroup closeness and impressions [18] or choice preferences [5,6]. In these previous studies, participants learnt differently from in-group and out-group individuals, indicated by a stronger effect for negative in-group experiences (negative in-group prediction errors [18]), and differential learning rates when learning from in-group compared with out-group experiences [5,6].

In contrast, in the current study, the strength of prediction-error-related updates in intergroup approach was mainly affected by the valence of the outcome and not the social group manipulation (indicated by the worse performance of the model differentiating between in-group and out-group learning rates). The observed differences in learning mechanisms are plausible in light of the most important difference in experimental manipulation. In the studies by Zhou *et al.* [18] and Traast *et al.* [5,6], positive and negative outcomes resulted from the decision of an in-group or out-group individual, i.e. the decision to help or not to help [18], or to share points with the participant [5,6]. Consequently, prediction errors and learning-related updating of participants’ behaviour resulted from actual in-group or out-group behaviour. In contrast, in the current study, participants received financial rewards or punishments that were unrelated to in-group or out-group behaviour. In consequence, the prediction-error-based updating of participants’ behaviour resulted from external feedback linked to the general valence of monetary reward or punishment. Hence, this domain-general reward successfully counteracted the positive in-group and the negative out-group prior based on the same mechanism for in-group and out-group.

Our finding that financial incentives can motivate out-group approach but have a limited effect on out-group impressions is of theoretical and practical relevance, because it highlights the limitation of financial incentives, i.e. the type of incentives that are frequently used to shape attitudes, motivation and behaviour. According to our findings, financial incentives can motivate people to approach out-groups but are unlikely to change negative out-group impressions. In other words, societal interventions that give people money to approach out-groups might seem successful based on observation of overt behaviour (because people approach the out-group more), but are probably not sufficient to change intergroup impression or beliefs. That said, increasing out-group approach based on learning from financial incentives is still relevant, because it can counteract perpetuation of avoidance behaviours towards out-groups (self-reinforced out-group avoidance; [19]) that is detrimental, because it prevents learning from positive experiences.

Our finding of participants showing higher learning rates for financial reward as compared with punishment is in line with previous literature demonstrating a positivity bias in learning from financial rewards [55–57]. In this literature, learning from positive learning rates was explained by participants seeking new opportunities primarily based on past successes, associated with a trade-off of exploration and exploitation in an exploratory context. However, in the reinforcement learning literature, higher learning rates for punishment are often reported as well [58,59]. For example, comparing positive and negative learning rates for different reward distributions, Gershman [60] could show consistently higher learning rates for negative reward prediction errors contrasting our results. They explain this tendency to learn more from negative feedback as a robust strategy to seek stability in a context of varying reward distributions in relation to risk aversion, which is rooted in evolutionary psychology. Participants in our approach–avoidance learning task started with a prior opposing the reward-optimizing behaviour. Our finding of a higher learning rate for positive prediction errors highlights the exploratory learning context with conflicting social preferences and demonstrates that participants understand the stability of the reward contingencies.

Based on the finding that participants mainly learnt from the positive and negative prediction errors without a difference in social groups (social conflict study), one could argue that participants simply acquired a positive or negative association with one of the symbols while discarding their social meaning. Our results show a significant in-group bias in impression ratings and in initial distance, indicating that participants associated the abstract symbols with different groups. However, it is still possible that the social meaning of the symbols fades away, and over time, participants start to learn only from the incentives. To control for this possibility, we implemented the social control study. The social control study is identical to the social conflict study, i.e. includes the same number of trials, the same abstract symbols that were associated with the in-group and the out-group and the same financial rewards and punishments. However, in contrast to the social conflict study, these rewards and punishments occurred randomly. The results of the social control study showed a significant in-group approach bias (figure 3) that was stable across blocks (trial × symbol × block interaction, *χ*^2^(1) = 0.16, *p* = 0.692, *β*_out-group_ = −0.01, s.e. = 0.02, *t* = −0.40). These results show that the social meaning of the symbols was maintained over time.

To further investigate the effect of the group manipulation on behaviour and learning, we designed the non-social control study. Both studies used the identical financial reinforcers and the identical experimental set-up. The only difference was the meaning of the symbols that were associated with the in-group and out-group in the social conflict study and had no social meaning in the non-social control study. If the observed changes in approach only reflect learning from financial incentives, the two studies should reveal comparable results. If the social meaning of the symbols alters behaviour and learning, the social conflict and the non-social control studies should show differential results. Supporting the latter, our findings show a significant trial × symbol × study interaction (*χ*^2^(1) = 9.53, *p* = 0.002, *β*_out-group, non-social control_ = −0.11, s.e. = 0.03, *t* = −3.09), indicating that learning-related changes in approach were different if the symbols were associated with the in-group and the out-group. Additional analyses revealed that the difference between the studies remained significant until the end of the task, showing that behaviour was still affected by the social meaning of the symbols (study × symbol interaction, last block (*χ*^2^(1) = 8.75, *p* = 0.003, *β* = −0.17, s.e. = 0.06, *t* = −2.96); last 10% of trials (*χ*^2^(1) = 7.33, *p* = 0.007, *β* = −0.28, s.e. = 0.10, *t* = −2.71); last 20% of trials (*χ*^2^(1) = 11.69, *p* < 0.001, *β* = −0.24, s.e. = 0.07, *t* = −3.42)).

Further evidence for the effects of the social group manipulation was provided by the computational modelling results. We found that changes in behaviour in the non-social study (i.e. behaviour towards symbols without a social meaning) were captured by a simpler model than learning-related changes in the social conflict study (i.e. towards symbols associated with the in-group and the out-group), indicated by the slightly lower BIC value for Model 1 compared with Model 5. That said, the best model for the social conflict study (Model 5) also performed fairly well in the non-social study, supporting the assumption that social and non-social learning share common mechanisms [61].

Our results were established using nationality (German versus Chinese), which is one of many possible social group manipulations. In line with previous studies [18,32,62–64], this social group manipulation induced significant effects, reflected in initial approach behaviour and the significant influence of individual in-group identification scores. Nevertheless, future studies may test if these results generalize to other intergroup settings. We conducted all three studies with independent online samples, which allowed us to collect data from diverse samples. Applying reinforcement learning modelling called for a standardized paradigm with a considerable number of trials [65]. Although we tried our best to make approach–avoidance decisions as intuitive and interesting as possible (participants could embody themselves in a manikin and then walk towards or away from a symbol associated with persons from the same or a different ethnicity), we acknowledge that our experimental set-up is artificial and the results should be replicated in other ecologically valid contexts. Moreover, participants might be less motivated during online data collection. Given that we nevertheless observed significant learning-related changes and differences in results between the studies in line with our *a priori* hypotheses, we are confident that the results of our online studies capture learning in a meaningful way. Future studies should test the stability of these learning effects without incentives and in different contexts.

In sum, our results show that financial rewards and punishments are well suited to induce learning-related changes in intergroup behaviour but are not likely to change intergroup impressions.

## Data Availability

All data are publicly available via the Open Science Framework [66]. All code for reproducing the analyses and figures is publicly available via the Open Science Framework [66]. Supplementary material is available online [67].
